# Integrated computational analyses reveal novel insights into the stromal microenvironment of SHH-subtype medulloblastoma

**DOI:** 10.1038/s41598-021-00244-3

**Published:** 2021-10-19

**Authors:** Alexander P. Landry, Nardin Samuel, Julian Spears, Zsolt Zador

**Affiliations:** Division of Neurosurgery, Department of Surgery, University of Toronto, Toronto, ON Canada

**Keywords:** Functional clustering, Gene regulatory networks, Network topology, Biomarkers, Cancer genetics

## Abstract

Medulloblastoma is the most common malignant brain tumour of childhood. While our understanding of this disease has progressed substantially in recent years, the role of tumour microenvironment remains unclear. Given the increasing role of microenvironment-targeted therapeutics in other cancers, this study was aimed at further exploring its role in medulloblastoma. Multiple computational techniques were used to analyze open-source bulk and single cell RNA seq data from primary samples derived from all subgroups of medulloblastoma. Gene expression is used to infer stromal subpopulations, and network-based approaches are used to identify potential therapeutic targets. Bulk data was obtained from 763 medulloblastoma samples and single cell data from an additional 7241 cells from 23 tumours. Independent bulk (285 tumours) and single cell (32,868 cells from 29 tumours) validation cohorts were used to verify results. The SHH subgroup was found to be enriched in stromal activity, including the epithelial-to-mesenchymal transition, while group 3 is comparatively stroma-suppressed. Several receptor and ligand candidates underlying this difference are identified which we find to correlate with metastatic potential of SHH medulloblastoma. Additionally, a biologically active gradient is detected within SHH medulloblastoma, from “stroma-active” to “stroma-suppressed” cells which may have relevance to targeted therapy. This study serves to further elucidate the role of the stromal microenvironment in SHH-subgroup medulloblastoma and identify novel treatment possibilities for this challenging disease.

## Introduction

Medulloblastoma is the most common malignant brain tumour in children, with an overall 5-year survival of approximately 70%. Once considered a single disease, it is now understood represent a collection of four molecularly and clinically distinct subgroups, each with its own set of subtypes^1–3^. The molecular, developmental, and clinical landscapes of these subgroups have been well characterized over the last several years, and important differences in outcome and therapeutic vulnerabilities have been identified. For example, patients with Wnt-subgroup tumours typically have excellent survival rates while the 5 year survival in group 3 is approximately 20–30%; the SHH subgroup intermediate survival outcomes and *TP53* mutations are associated with a higher risk phenotype^2,4^. The role of tumor microenvironment (TME), defined as the interactions between neoplastic and non-neoplastic cells, has received increasing attention from the oncology community for its ability to shape tumour biology and yield important therapeutic vulnerabilities^5^. However, its role in medulloblastoma has yet to be as comprehensively explored.

The study of TME has led to highly effective therapeutic options, such novel immunotherapies for melanoma^6^, lung cancer^7^ and hematological malignancies^8^. To date, our understanding of the immune and stromal composition in the medulloblastoma microenvironment remains in its early stages, though early evidence suggests key subgroup-specific signatures^9^. In particular, the SHH subtype appears to be enriched in both immune and stromal fractions and its constituent tumor-associated macrophages seem to play an anti-tumor role^10^. Additionally, placental growth factor has been proposed as a key mediator of tumor-stroma interactions in medulloblastoma with possible therapeutic appeal, though its subgroup-specific role is unclear^11^. In this exploratory study, we apply established computational methods to well-annotated bulk and single cell medulloblastoma transcriptomic data in order to better define its stromal microenvironment and identify targetable pathways with therapeutic potential.

## Methods

### Data processing

Bulk data was obtained from the open repository *Gene Expression Omnibus*^12^ under the accessions GSE85217^2^ and GSE37382^13^, the latter was used as a validation cohort. Raw expression data was imported, background-corrected and quantile normalized with RMA, and log2 transformed before being used for subsequent analysis. Single cell data was obtained using the same platform, under the accessions GSE119926^1^ and GSE155446^14^, the latter again being used as a single cell validation cohort. Expression data was normalized and scaled using the default parameters in the *SEURAT*^15,16^ pipeline*.* Specifically, feature counts for each cell are divided by the cell’s total counts and multiplied by a scale factor (10,000, as default), natural log-transformed, then scaled and centered. Details on data procurement from original samples can be found in the cited publications.

### Bulk purity analysis

We applied the previously established tool ESTIMATE^17^ to bulk RNA expression data in order to extrapolate relative proportions of neoplastic, immune, and stromal cells for each tumour. All settings were kept as default. Briefly, this method compares expression data to “stroma” and “immune” signatures previously derived and validated on several tumor types. The proportion of neoplastic cells (labeled the “purity” of a tumour) was shown to correlate well with DNA copy number variation analysis. Stromal, and immune scores were compared between the four molecular subgroups, and 12 subtypes, of medulloblastoma using ANOVA and individual comparisons were performed with a t-test. Throughout the study, we consider a *p*-value < 0.05 to be significant.

### Receptor/ligand analysis

We used the FANTOM^18^ consortium of known receptor-ligand interactions to identify targetable extracellular molecules that may play a role in tumour microenvironment through cellular cross-talk between neoplastic and non-neoplastic cells. The details of this repository are described elsewhere in detail^18^. Briefly, the receptor-ligand interaction database was generated through the multi-step filtering of human protein-coding genes (HGNC) and extension of the existing databases Database of Ligand Receptor Partners (DLRP)^19^, IUPHAR^20^ and Human Plasma Membrane Receptome^21^. Receptor-ligand pairs were established by cell types and incorporated into an interactome model which allows the inference of interactions between two or even multiple cells^22,23^. In our analysis, we firstly defined the list of genes upregulated in medulloblastoma subgroups/single cell clusters then filtered these genes using FANTOM. This yielded a list of potentially targetable receptor-ligand pairs which underlie a particular phenotype.

### Differential gene analysis

In order to identify subgroup-specific drivers of stroma abundance, we applied differential gene expression (DGE) to ligands and receptors separately for relevant subgroups. Each gene was correlated to stroma score with a Pearson test; genes with absolute fold-change > 2 and *p* < 0.05 in the DGE and absolute correlation > 0.2 and *p* < 0.05 in Pearson correlation were selected as subgroup-specific “stroma drivers” or “stroma suppressors”, depending on their correlation to stroma score. Using the same approach, we explored the subgroup-specific correlations to stromal score for each identified receptor and ligand to identify differences in TME influence by subgroup.

### WGCNA

Weighted Gene Co-Expression Analysis (WGCNA)^24,25^ is a computational method used to identify co-expressed genes subserving a common biological function, a contrast to the more traditional single-gene approach to transcriptomic analysis. This both reduces the stochasticity associated with single-gene analysis and may yield new insights into a biological system, since meaningful biologic changes may be incurred by small perturbations in the expression of several genes along the same pathway^26–29^. Briefly, highly co-expressed genes are grouped into “modules”, whose overall expression is described by the value of the first principal component of the expression of its constituent genes, an established procedure. We applied WGCNA to bulk RNA-seq data using default parameters. The expression of select modules was also evaluated in the single cell data. Module function was annotated using the gene-enrichment tool *Enrichr*^30,31^*.*

### Network analysis

Receptors and ligands of interest were visualized as networks using Cytoscape^32^ for both bulk and single cell data. Nodes represent genes and edges weights correspond to Pearson correlations. Node size is made proportional to its closeness centrality, a measurement of its proximity to other nodes within a graph. Briefly, this assigns a numeric score to each node within a network which is inversely proportional to its average distance to all other nodes; thus, high closeness centrality is associates with the ability to propagate information throughout a graph with high efficiency. Therefore, nodes with the highest closeness centrality within a gene network are likely to portend significant influence on the overall output of the network.

### Single cell analysis

Single cell transcriptomic analysis relied on the well-established *SEURAT* pipeline. After preprocessing (described above), *Uniform Manifold Approximation and Projection (UMAP)*^33^ was used to plot and cluster the data in 2 dimensions with default parameters. Subgroup-specific ligand/receptor density was assessed with the same embedding, and cells with expression of many ligands/receptors were compared to those with few (exact cutoffs are subgroup-specific) using the *FindAllMarkers* function in Seurat (default parameters). A group’s list of defining genes (marker genes) was taken as all upregulated genes with an adjusted *p*-value of < 0.05, Marker genes were then annotated using the gene-enrichment platform *Enrichr*^30,31^ to characterize a group’s function*,* and relevant transcription factors/pathways (adjusted *p* < 0.05) were labelled*.*

### Inference of cell developmental potential

In order to assess single cells for developmental potential, we implemented the recently developed *CytoTRACE* pipeline^34^. Briefly, this approach numerically defines the “developmental potential” (i.e. stemness) of a cell based on the number and functional diversity of the genes it expresses. This has been extensively validated against ground truth cell states and outperforms other comparable methods. We further verified these results using a complementary approach that computes validated gene signature scores^35^, where “stemness” is calculated as the scaled average expression of genes within the signature minus the scaled average expression of the remaining genes.

### Computational analysis

All analyses in this paper were performed using the openly available statistical software R^36^ (version 3.6.3).

## Results

### Patient demographics

The bulk discovery dataset contains 763 primary medulloblastoma tumours. The mean age of patients at the time of sample acquisition is 10.4 years (SD 9.4 years) and 62% are male. The mean survival is 4.9 years (SD 3.8 years) from diagnosis. Patients are divided into group 3 (n = 144, 18.8%), group 4 (n = 326, 42.7%), SHH (n = 223, 29.2%), and Wnt (n = 70, 9.2%) molecular subtypes. Of 573 patients with metastatic status annotated, 176 (30.7%) have metastatic tumours. Specifically, metastases are present in 43/109 group 3 tumours (39.4%), 101/255 group 4 tumours (39.6%), 26/160 SHH tumours (16.3%), and 6/49 WNT tumours (12.2%). The bulk validation cohort has 285 primary medulloblastoma tumours. Mean age is 9.0 years (SD 5.9 years) and 72% are male. No tumours belong to the WNT subgroup; 51 (17.9%) are SHH, 46 (16.1%) are group 3, and 188 (66.0%) are group 4. Outcome data is not available in this cohort.

In the single cell discovery data, 7241 cells were amalgamated from 23 tumours, with a mean of 315 cells per tumor (range 52–507). Cells were divided into 7 group 3 tumours (n = 2448, 33.8%), 8 group 4 tumours (n = 1903, 26.3%), 3 SHH tumours (n = 1124, 15.5%), and 5 WNT tumours (n = 1766, 24.4%) using the authors labels. In this cohort, 14/23 (60.9%) of tumours have metastases, corresponding to 64% of total cells. Specifically, metastatic disease is present in 5/7 (71.4%) of group 3 tumours, 4/8 (50.0%) of group 4 tumours, 2/3 (66.7%) of SHH tumours, and 3/5 (60%) of WNT tumours. In the single cell validation cohort, 32,868 cells were amalgamated from 29 tumours, divided into 6 group 3 tumours (n = 10,175, 31.0%), 1 group 3/4 tumour (n = 2420, 7.4%), 12 group 4 tumours (n = 13,491, 41.1%), 9 SHH tumours (n = 6044, 18.4%), and 1 WNT tumour (n = 738, 2.2%).

### SHH-subtype medulloblastoma is enriched in stromal signature

**Figure 1:**
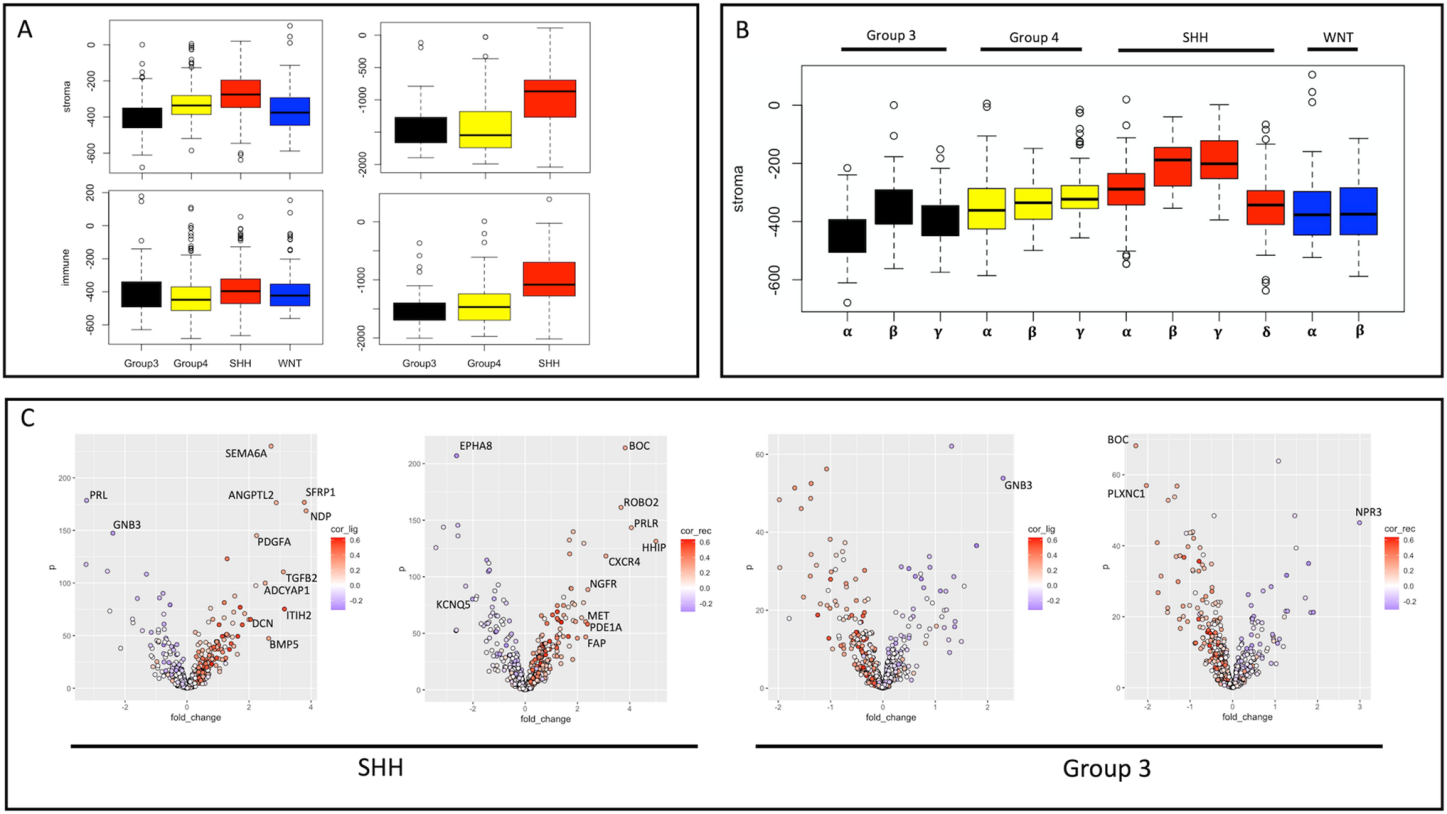
Stromal microenvironment is enriched in SHH-subtype medulloblastoma and suppressed in group 3. (**A**) ESTIMATE calculation of stroma (upper) and immune (lower) scores by molecular subgroup. Discovery cohort is plotted on the left and validation cohort is on the right. (**B**) Boxplot of stromal score by molecular subtype (ANOVA *p* < 0.05). (**C**) Volcano plots of differentially expressed ligand (left) and receptor (right) genes for SHH (stroma-upregulated) and group 3 (stroma-downregulated). For each subgroup, comparison is made to the pooled amalgamation of the remaining 3 subgroups. Each receptor/ligand gene is coloured based on its correlation with stromal signature score, with red indicating positive correlation and blue indicating negative correlation. Those with absolute fold-change > 2 and absolute Pearson correlation > 0.2 (labeled) are selected as potential drivers of subgroup-specific stromal differences (drivers of stroma signature in SHH-subgroup and repressors of stromal signature in group 3).

We firstly explored the subgroup-specific differences in neoplastic, stroma, and immune composition of medulloblastoma using ESTIMATE^17^, a well-established algorithm for assessing TME using bulk transcriptomics. We found statistically significant (ANOVA *p* < 0.05) differences between medulloblastoma subgroups, with the greatest effect size noted in the stromal population (Fig. 1A, left). Notably, the SHH-subgroup is significantly enriched in its stromal population compared to all other subgroups, while group 3’s stromal score is significantly suppressed compared to each other subgroup, which remains true in the validation cohort (Fig. 1A, right). We therefore focus on the SHH (“stroma-enriched”) and group 3 (“stroma-suppressed”) subgroups in subsequent analysis of tumor-stroma interactions in medulloblastoma. We further explored the above characteristics of stromal microenvironment in the more refined molecular subclasses of medulloblastoma defined through the integration of multimodal genomic data (Cavalli et al.^2^). Within this integrated classification, the four major medulloblastoma subgroups are refined to 12 subtypes. We find the highest stromal scores in SHH subtypes beta and gamma (Fig. 1B). By contrast, group 3 alpha is the most stroma suppressed.

### Distinct receptor-ligand interactions associated with SHH medulloblastoma subtype

**Figure 2:**
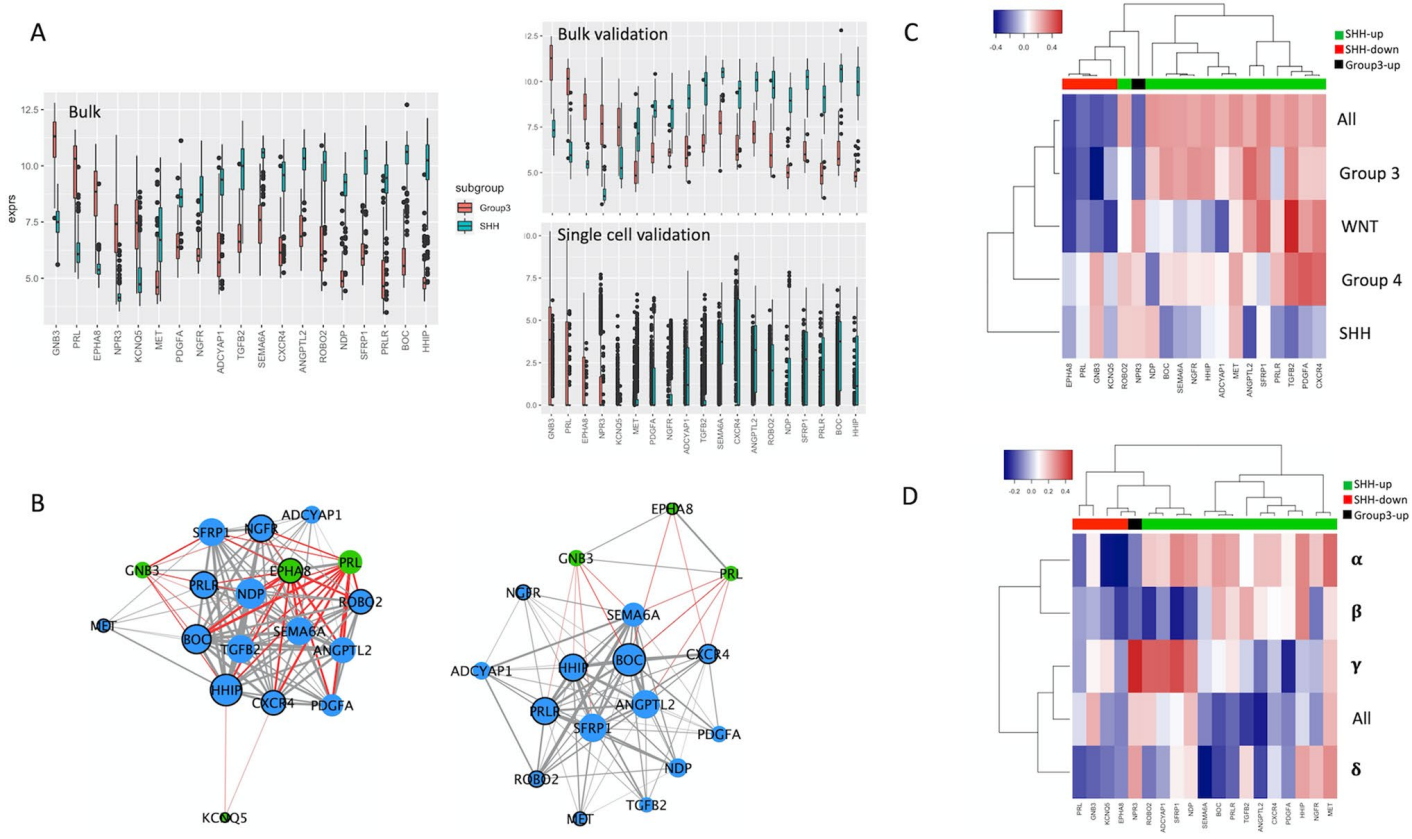
Characterizing stroma-associated receptors/ligands in bulk and single cell data. (**A**) Boxplots comparing the expression of all receptors/ligands of interest (stroma-associates) between SHH and Group 3 medulloblastoma. Genes expressed in < 1% of single cells are excluded, as are genes in the stromal signature panel. Discovery cohort is plotted on the left, bulk validation is right upper and single cell data is right lower. (**B**) Network analysis of genes from (**C**) in bulk (left) and single cell (right) data. Edge thickness is proportional to absolute Pearson correlation (minimum 0.6 in bulk, 0.2 in single cell, all *p* < 0.05), with red lines indicating negative correlation. Node size correlates with its closeness centrality. Blue nodes represent SHH-upregulated/Group 3-downregulated/stroma-correlated genes, whereas green nodes represent Group3-upregulated/SHH-downregulated/stroma-anticorrelated genes. Nodes with borders are receptors and those without are ligands. C: Heatmap indicating subgroup-specific Pearson correlations between stromal score and selected stroma-associated genes from Fig. 1. Colour bar above represents correlation of receptor/ligand gene expression with subtype label. Note that GNB3 is both SHH-downregulated and Group 3-upregulated, while the reciprocal is true of BOC. (**D**) Heatmap indicating SHH subtype-specific Pearson correlations between stromal score and stroma-associated genes. Colour bar as in C.

We next attempted to identify receptor-ligand interactions associated with the distinct stromal compositions of SHH and group 3 tumours, which may serve as targetable neoplastic-stromal communication pathways. We therefore investigated the expression patterns of known receptors and ligands from the FANTOM consortium. We found that subgroups separated well based on both receptor and ligand expression patterns using t-SNE representation (Fig. 1B). Differential gene expression identifies several SHH and group 3-specific receptors/ligands which correlate to stromal score (Fig. 1C). Interestingly, the majority of genes upregulated in SHH medulloblastoma correlate positively with stroma score and those upregulated in group 3 medulloblastoma correlate negatively to stroma score (the inverse is also true). This suggests that differences in stromal signature may be manifested through several differentially-expressed extracellular signaling mechanisms, a redundancy that will be important to consider when designing therapeutics. We define *SHH-stroma-drivers* as ligands/receptors which are positively correlated to stroma score and either upregulated in SHH-subgroup or downregulated in group 3 subgroup (cutoff fold-change 2, Pearson correlation 0.2, *p* < 0.05). By contrast, *group3-stroma-suppressors* are ligands/receptors which are negatively correlated with stroma score and either upregulated in group 3 or downregulated in SHH subgroup. For clarity, we will refer to the collection of *SHH-stroma-drivers* and *group3-stroma-suppressors* as *stroma-associates*. As illustrated in Fig. 2A, strong differences are found in the expression of these *stroma associates,* and the relationships are held both in bulk validation and in single cell data. To explore the pairwise interactions within the group of *stroma-associates,* we carried out a network analysis in bulk and single cell data separately. This suggests a consistent central role of SHH-upregulated receptors HHIP, BOC, PRLR, CXCR4 and ligands SFRP1, SEMA6A, and ANGPTL2 (Fig. 2B). Notably, each *stroma-associate*’s correlation with stroma score is subgroup-specific (Fig. 2C), suggesting that one must consider its context and expression in each subgroup individually. Furthermore, correlation with stroma score is also subtype-specific within the SHH subgroup itself as illustrated in Fig. 2D.

### WGCNA further supports differences in TME between subgroups

**Figure 3:**
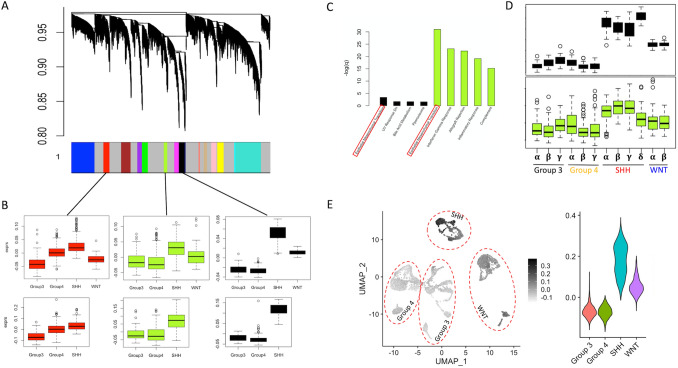
Weighted gene co-expression network analysis (WGCNA) yields SHH-specific TME signatures from the whole transcriptome. (**A**) Dendrogram depicting gene clustering and module selection, where module groupings are annotated by the color bar below. (**B**) Boxplots of the meta-gene expression for each module with highest expression in the SHH subgroup. Computed meta-gene expression of the same modules in the bulk validation cohort are present below. (**C**): Module annotation using MSigDB Hallmark genes. Only functions with adjusted *p* < 0.05 are included, and no more than 5 are included for each module. (**D**) Meta-gene expression of the black and greenyellow modules by molecular subtype in the discovery cohort. Note that the black module is strongly upregulated in all 4 SHH subtypes. E: UMAP plot of single-cell data, coloured by the meta-gene expression of the black module. The adjacent violin plot demonstrates significant upregulation in the SHH subgroup, which is consistent with bulk results.

Next, we used a weighted gene co-expression network analysis (WGCNA) as a complementary approach to confirm ligand/receptor associates of SHH (“stroma-enriched”) and group 3 (“stroma-supressed”) phenotypes. We apply WGCNA to all genes in the bulk data, yielding 15 co-expression modules ranging in size from 115 to 2597 genes (Fig. 3A). Three modules with highest median meta-gene expression in the SHH subgroup (ANOVA *p* < 0.05) were extracted for further analysis, and their computed values in the validation cohort matched the relationships found in the discovery cohort (Fig. 3B). Annotation of these modules identified two modules with strong enrichment with the process of “epithelial to mesenchymal transition”, a phenotype transition widely observed in solid neoplasia and associated with invasion and metastasis^37^ (Fig. 3C). Analysis stratified by subtype demonstrates that one module in particular is upregulated strongly in all SHH subtypes (Fig. 3D). This module is also strongly upregulated in the SHH subgroup in single cell data (Fig. 3E), as did known epithelial-to-mesenchymal marker genes ZEB1, TWIST2, and SOX9 (Supplemental Fig. 1).

### Single cell analysis identifies expression gradient underlying TME in SHH-medulloblastoma

**Figure 4:**
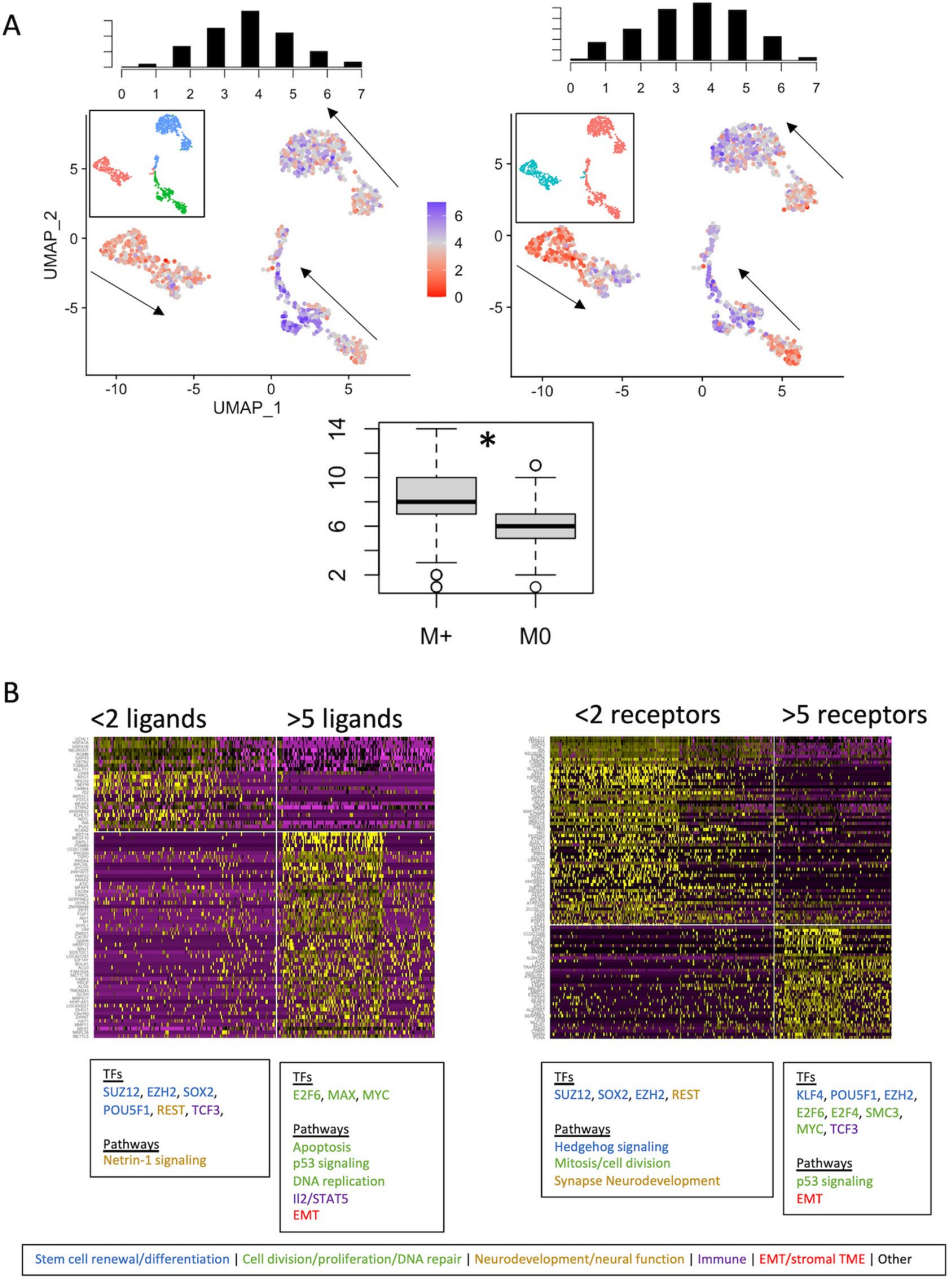
Characterization of stroma-associated single cell programs in SHH subtype. (**A**) UMAP plots of single cell transcriptomic data from SHH medulloblastoma. Cells are coloured based on the number of stroma-associated ligands (left) or receptors (right), as identified in Fig. 2. Above histograms represent the respective associated frequency distributions of ligand/receptor expression per cell. Inset images are the same UMAP plot, coloured by tumor ID (left) and metastatic status (right; red = metastasis present). We note apparent gradients from low to high density within each individual tumour, which is emphasized by arrows. (**B**) Markers associated with low vs. high number of ligands (left) or receptors (right) expressed (labeled above). Yellow indicates higher expression, while purple indicates low expression. Key enriched transcription factors (TFs) and pathways (only those with corrected *p* < 0.05) are labeled below and coloured by function. We note a predominance of developmental programs in cells with few stroma-associated ligands/receptors, while those which express more such receptors/ligands appear to be enriched in cell-cycling, EMT, and immune function.

We next investigated the cell-level gene expression programs associated with the distinct TME phenotypes observed in SHH medulloblastoma (Fig. 4A). Firstly, independent UMAP clustering shows considerable intertumoral heterogeneity, such that each tumour is defined by a distinct cluster. We next investigate intratumoral heterogeneity and its relation to stromal interactions by investigating the number of subgroup-specific *stroma-associates* expressed by each cell. In the SHH subgroup, there appears to be a preserved biological gradient within each tumour, evolving from cells expressing few *SHH-stroma-drivers* to those expressing many (Fig. 4A). Given that this pattern is observed within the data topology of UMAP clusters (a function of overall gene expression), this suggests fundamental differences in biology extending beyond the number of these *stroma-associates* expressed. Additionally, metastatic SHH-tumours are upregulated in *SHH-stroma-drivers* compared to non-metastatic tumours (Fig. 4A, *p* < 0.05), suggesting that “stroma-active” SHH-medulloblastoma cells play a role in metastatic spread.

We next investigated the gene expression programs associated with higher ligand/receptor abundance by comparing “stroma-active” SHH cells (those expressing > 5 *SHH-stroma-drivers*) to “stroma-inactive” SHH cells (those expressing < 2 *SHH-stroma-drivers*) (Fig. 4B). Marker genes defining “stroma-inactive” cells subserve functions predominantly related to stemness/development with specificity toward neurodevelopment and neural function. In comparison, marker genes defining “stroma-active” cells also map onto developmental pathways with predilection for cell cycle functions as well as the epithelial to mesenchymal transition and immune development. From these results, we conclude that the SHH subgroup carries a spectrum of biology on the single cell level which is intimately associated with its stroma interactions. The apparently crucial role that the density of *stroma-associates* plays in the topology of single cell data is independently validated, as shown in Supplemental Fig. 2.

**Figure 5:**
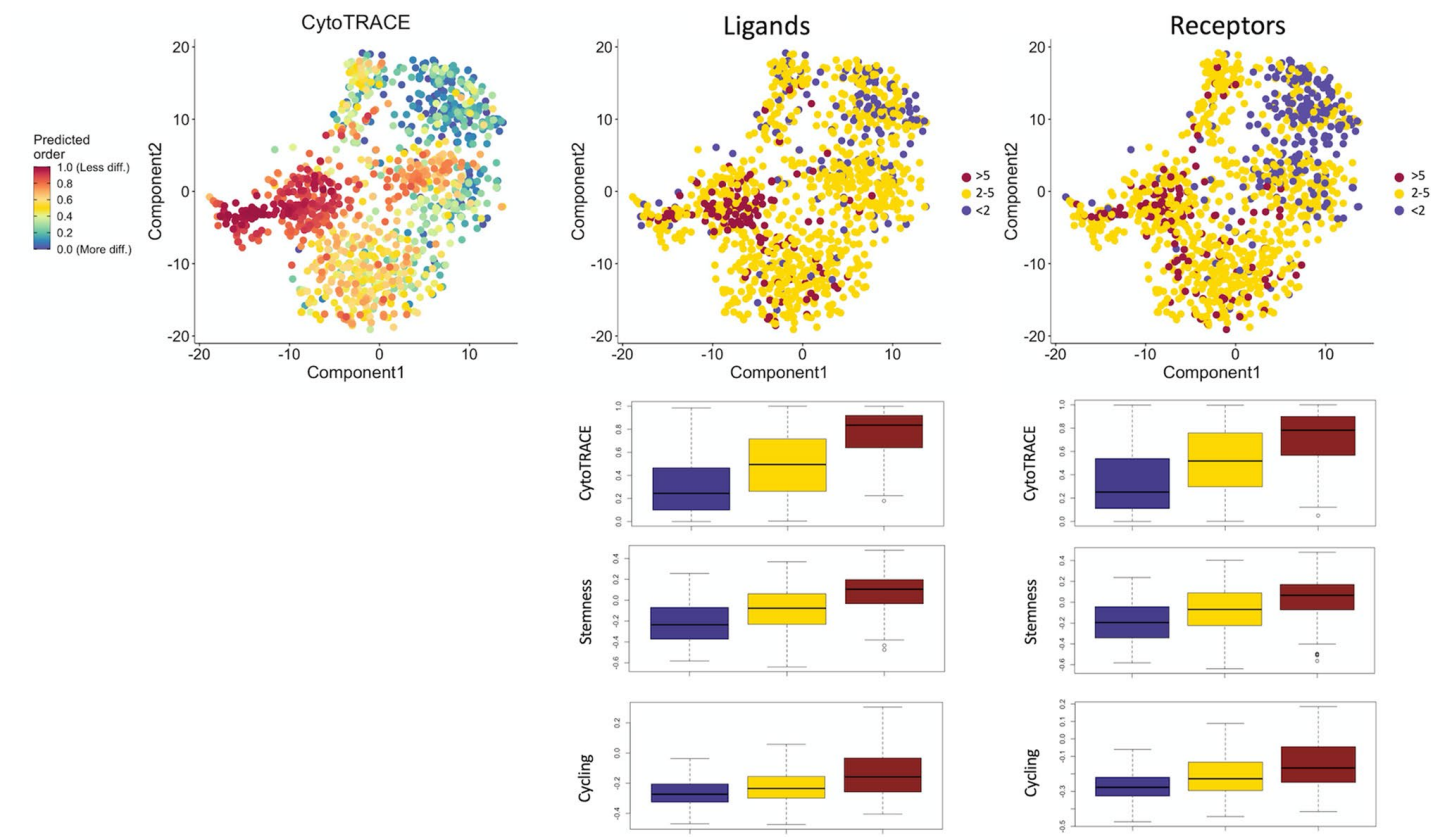
Establishing the developmental hierarchy of SHH medulloblastoma as a function of stromal activation. Top-left: t-SNE embedding of CYTOTRACE output, where less differentiated cells are coloured red and more differentiated cells are coloured blue. The same embedding is coloured by ligand (top-middle) and receptor (top-right) density of stroma-associates, suggesting “stroma-active” cells have greater developmental potential than “stroma-inactive” cells. Boxplots are used to compare stemness and cycling properties of cells expressing < 2, 2–5, and > 5 receptors and ligands, using both CYTOTRACE output and previously established “stemness” and “cycling” gene signatures. ANOVA *p* < 0.0001 in all cases.

Previous studies have demonstrated that intratumoral heterogeny is due partly to a distribution along a developmental hierarchy^35^, which can be characterized based on each cells “differentiation potential”. Importantly, we find a positive correlation between a SHH-subgroup cell’s developmental potential and the number of *SHH-stroma-drivers* it expresses (Fig. 5, Pearson correlation 0.54, *p* < 0.0001). We also find that cells expressing more *SHH-stroma-drivers* are enriched in signatures related to cell cycling and stemness^38^. These findings are replicated in Supplemental Fig. 3 using the single cell validation cohort. In sum, we suggest that SHH-subgroup medulloblastoma exhibits preserved intratumoral heterogeneity marked by a developmental gradient from “stroma-active” cells, characterized by high expression of *SHH-stroma-drivers*, high developmental potential, cycling cells, and enrichment of the EMT program to “stroma-inactive” cells, characterized by low expression of *SHH-stroma drivers,* less developmental potential, and enrichment of neurodevelopmental programs. Importantly, a greater number of “stroma-active” cells may increase risk of invasion and metastasis, and *SHH-stroma-drivers* may represent key therapeutic vulnerabilities of this medulloblastoma subgroup.

## Discussion

The findings in this study demonstrate significant differences in the tumor microenvironment between medulloblastoma subgroups, with the SHH-subgroup enriched in the stromal population while group 3 is comparatively stroma-suppressed. We identify several ligands and receptors which drive this phenotype in the SHH (labeled *SHH-stroma-drivers*) and group 3 (labeled *group3-stroma-suppressors)* which may represent subgroup-specific therapeutic targets. We also find the epithelial-to-mesenchymal pathway, an important stroma-associated cancer target, to be enriched in the SHH-subgroup using multiple approaches. Single-cell expression data in SHH medulloblastoma suggests a biological gradient from “stroma-active” cells expressing several SHH-stroma-drivers towards a “stroma-inactive” phenotype. Notably the “stroma-active” phenotype is less differentiated and is enriched in the epithelial to mesenchymal transition and immune development while “stroma-inactive” cells, with fewer *SHH-stroma-drivers*, have less developmental potential and enrich in functions related to neurodevelopment. The stroma-suppressed state of group 3 medulloblastomas, and neurodevelopmental dependencies of “stroma-inactive” cells, suggests that unlike in the case of the SHH subgroup, their aggressiveness is not a function of microenvironment and that different treatment strategies should be sought.

### The role of stroma in cancer biology

The role of tumour microenvironment in cancer biology has received increasing attention in recent years, related largely to the profound impact of immune-targeted therapies in cancers such as melanoma and non-small cell lung cancer. While immunotherapy in neuro-oncology remains in its infancy, we remain hopeful that the ongoing research efforts will prove fruitful in the near future. The investigation of tumour-stroma interactions represents another exciting avenue through which cancer may be targeted, though we continue to await the development of stroma-specific therapies. Importantly, the quest to subdue the most treatment-resistant of tumour cells is likely to require a multi-pronged strategy, especially given the long history of failed clinical trials in neuro-oncology. To this end, targeting the stromal fraction of TME may represent another avenue through which the tumour “ecosystem” can be disrupted. Importantly, stroma cells in TME are known to play key tumour-modulating roles and to promote chemoresistance, making them attractive (albeit complex) therapeutic targets^39^.

The epithelial to mesenchymal transition (EMT), which was strongly associated with the SHH-subgroup in this study, is crucial in the evolution of TME. In essence, the EMT is the process through which epithelial cells detach from their extracellular scaffolding and mesenchymal programs are subsequently activated, resulting in a graded evolution of cell characteristics. The process is critical in embryogenesis and wound healing, and in cancer is associated with increased metastatic potential and chemoresistance^40–42^. This has been shown to hold true in SHH-medulloblastoma as well, wherein the transition is proposed to be mediated by VEGFA-NPR2 signalling^43,44^. This suggests EMT as a potentially targetable SHH-enriched driver of metastasis which warrants further study.

### SHH-subgroup-drivers may represent therapeutic targets in medulloblastomas

Several of the stroma-associated ligands and receptors identified in this study have previously been shown to be promising targets in SHH-subgroup medulloblastoma. Platelet derived growth factor A (PDGFA) has been shown to correlate with metastasis and migration in SHH cell lines, and PDGFRA inhibition was able to block PDFGA-mediated migration^45^. CXCR4 is an important chemokine receptor in cerebellar development and interacts with SHH signaling pathways. It is known to play a role in tumor microenvironment and is implicated in SHH medulloblastoma tumorigenesis; importantly, its inhibition has shown promise in medulloblastoma therapy^46^. BOC is a driver of SHH signaling and is associated with granule cell precursor proliferation and medulloblastoma tumorigenesis; inactivation is associated with decreased medulloblastoma progression^47^. Similarly, MET is a marker of metastasis in SHH medulloblastoma, and it’s inhibition has been shown to decrease proliferation and induce tumor cell apoptosis^48^. NGFR (CD271) is a marker of SHH medulloblastoma, and CD271^+^ medulloblastoma cells exhibit increased Ras/MAPK signaling, invasion, and migration which can be successfully targeted with MEK inhibition. Paradoxically, low CD271 expression is correlated with worse outcome, which may indicate a slower cycling but treatment-resistant phenotype^49^. Overall, inhibition PDGFA, CXCR4, BOC, MET, and NGFR have all shown therapeutic promise in SHH-subgroup medulloblastoma.

While many tumour-stroma interactions appear to drive invasion and metastasis, this communication is complex and bidirectional; some of the *SHH-stroma-drivers* identified in this study have been negatively associated with tumorigenesis and proliferation, though not specifically with metastasis. Blockage of Norrin, the protein product of the NDP gene, in SHH medulloblastoma has been shown to create a tumor-permissive, stromal-driven microenvironment^50^. PACAP, the product of ADCYAP1, is involved in the regulation of granule neuron precursor proliferation (the putative cell-of-origin of SHH medulloblastoma) and has been shown to antagonize Hedgehog signaling and reduce proliferation in SHH MB tumorspheres^51^. Similarly, HHIP reduces stromal-associated malignancy via inhibition of HH signaling^52^. These results show that TME interactions are highly complex, and the influence of each receptor-ligand interaction on a tumour ecosystem must be carefully considered.

The effects of the remainder of the *SHH-stroma-drivers* on medulloblastoma are less clear. Transforming growth factor beta (TGF-B) signaling is known to antagonize the SHH pathway in medulloblastoma and has been shown to modulate invasiveness in a context-dependent fashion^53^. SFRP1, a WNT inhibitor and marker of SHH medulloblastoma, is a downregulated tumour suppressor in all other subgroups; it’s role within the SHH subgroup is sunclear^54^. SEMA6A is involved in neuronal differentiation and has also been shown to be a marker of SHH medulloblastoma^1^, though exhibits tumor suppressive effects in groups 3 and 4^55^. ROBO2 is part of the SLIT/ROBO complexes, which plays a role in neural and vascular development^56^. They may act as tumor suppressors or oncogenes, depending on the context, though the specific role of ROBO2 in medulloblastoma is unclear. The prolactin receptor (PRLR) was shown to play an intimate role in a mouse model of cancer-fibroblast interactions by mediating micrometastases via COX-2; COX-2 inhibition was shown to reduce tumorigenesis^57^. It’s role in medulloblastoma is unclear. Similarly, in a bioinformatics-driven model of gene expression in medulloblastoma, ANGPTL2 is proposed as one of several possible driver copy number alterations of the SHH subgroup, though further confirmation is necessary to better understand its role^58^. It is involved in tissue repair and angiogenesis and may function as either a tumor promotor or inhibitor, depending on the cancer^59,60^. In summary, many *SHH-stroma-drivers* identified in this study are known to play key roles in TME; those whose role in medulloblastoma have yet to be elucidated warrant further in-depth investigation.

### Limitations and future directions

Some study limitations should be noted. Firstly, the datasets utilized in this study are derived from prior studies and thereby limit our ability to compare datasets given differences in data procurement and processing. Some samples from the single cell data have been enriched in neoplastic cells through counter-selection for CD45 marker, limiting the ability to directly assess cellular cross-talk between malignant and non-malignant cells. In addition, we do not have complete clinical annotation of the single cell data, which may have allowed for more comprehensive analysis. Nevertheless, the objective of this work was to use computational techniques in order to identify novel avenues for therapy which can subsequently be tested in more rigorous fashion.

Given these identified receptors and ligands, we would hope to target them in subgroup-specific cell lines and/or animal models to assess for clinical viability. This may lead to novel clinical trials targeting stromal microenvironment of medulloblastoma.

## Conclusions

This study is among the first to investigate subgroup-specific stromal TME in medulloblastoma. By applying computational methods to bulk and single cell data, we find the SHH-subgroup to be stroma-enriched, whereas group 3 tumours are relatively stroma-suppressed. We identify multiple receptor-ligand drivers of these phenotypes, some of which may yield novel therapeutic targets. Single cell transcriptomics reveals important intratumoral dynamics within the SHH subgroup, which associate intimately with the expression of stroma drivers. We propose the notion that the “stroma-active” SHH tumour cells play a key role in recurrence, and that identifying their vulnerabilities will be important to controlling this challenging and heterogeneous disease. The successful treatment of highly resistant medulloblastoma cells may ultimately require a subgroup-specific multi-pronged approach which targets cancer cells, stromal cells, and immune cells simultaneously. Future studies should be aimed at further investigating the candidate therapeutic targets identified in this study.

## Supplementary Information

Supplementary Information.
