# Supplementary material for: Integrated computational analyses reveal novel insights into the stromal microenvironment of SHH-subtype medulloblastoma
Source: Sci Rep. 2021 Oct 19;11:20694. doi: 10.1038/s41598-021-00244-3 (PMC8526813; doi:10.1038/s41598-021-00244-3)
Supplement: Supplementary file 1 — Supplementary Information. [file 41598_2021_244_MOESM1_ESM.pdf]

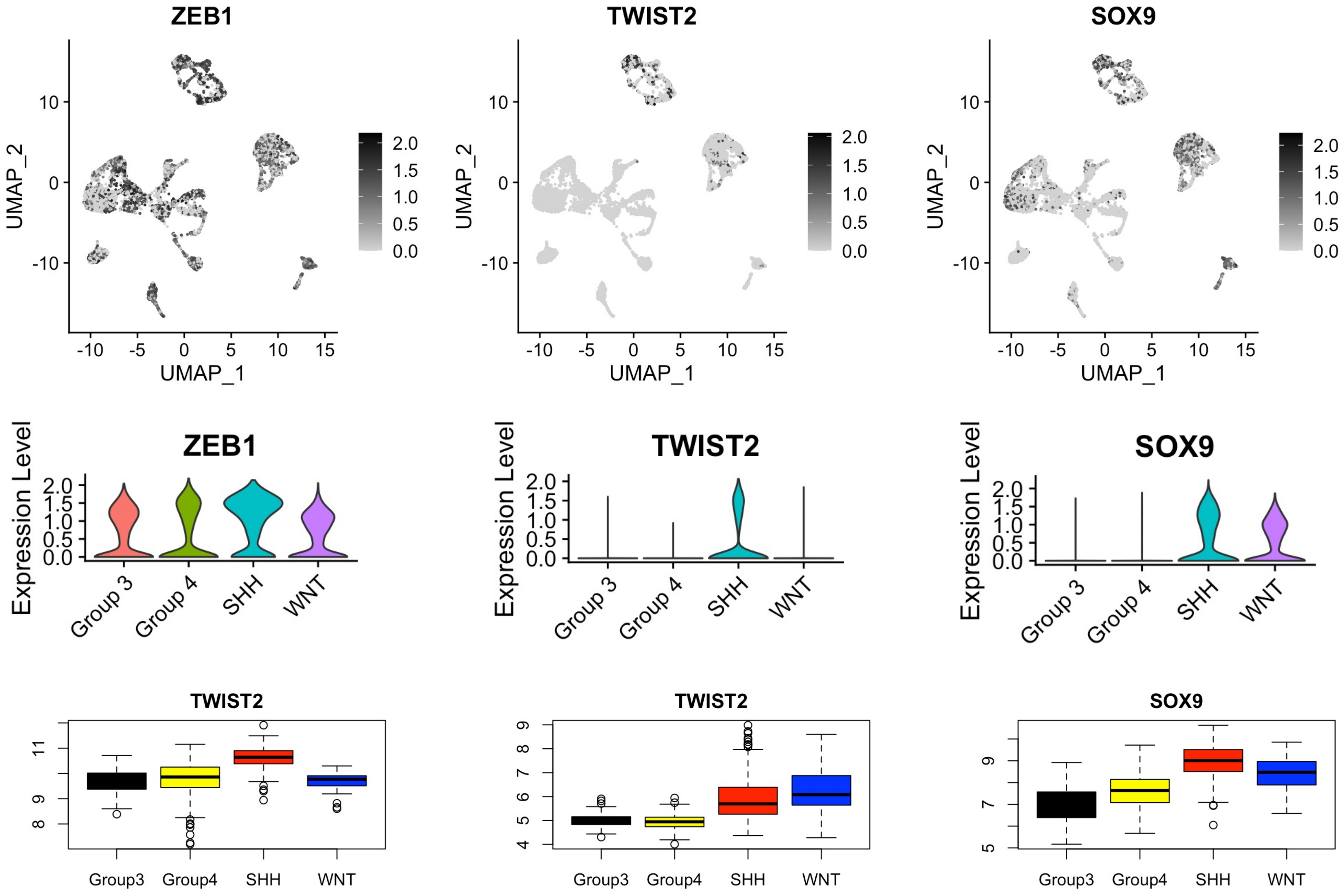

Supplemental Figure 1: Expression of EMT markers by SHH subgroup. Top row: expression by UMAP clusters. Middle row: subgroup-specific expression in single cell data. Bottom row: subgroup-specific expression in bulk data.

Supplemental Figure 2: Validation of stroma-associated single cell programs in SHH subtype. A: UMAP plots of single cell transcriptomic data from SHH medulloblastoma. Cells are coloured based on the number of stroma-associated ligands (left) or receptors (right), as identified in Figure 2. Inset images is the same UMAP plot, coloured by tumor ID. B: Markers associated with low vs. high number of ligands (left) or receptors (right) expressed (labeled above). Yellow indicates higher expression, while purple indicates low expression. Key enriched transcription factors (TFs) and pathways (only those with corrected p<0.05) are labeled below and coloured by function.

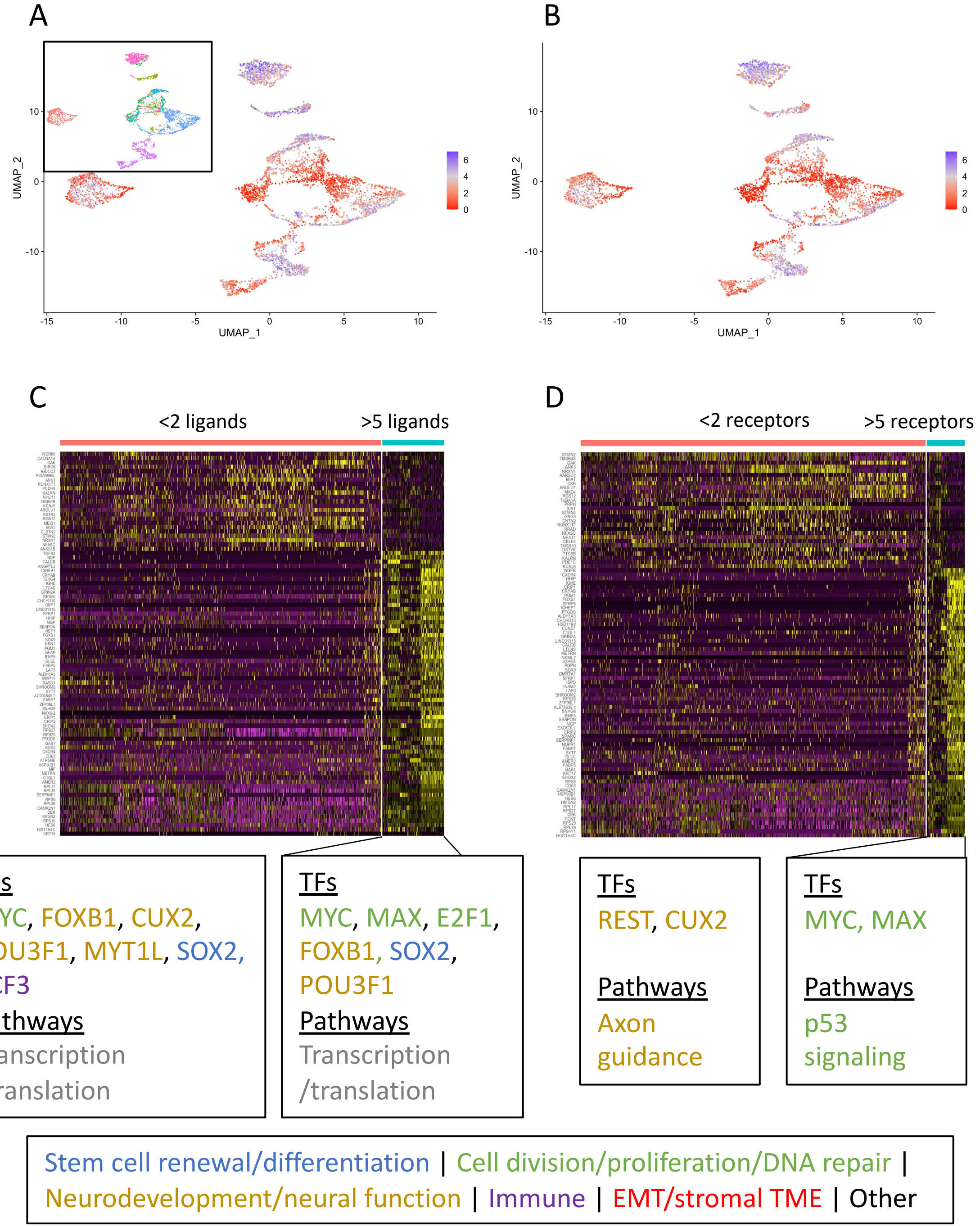

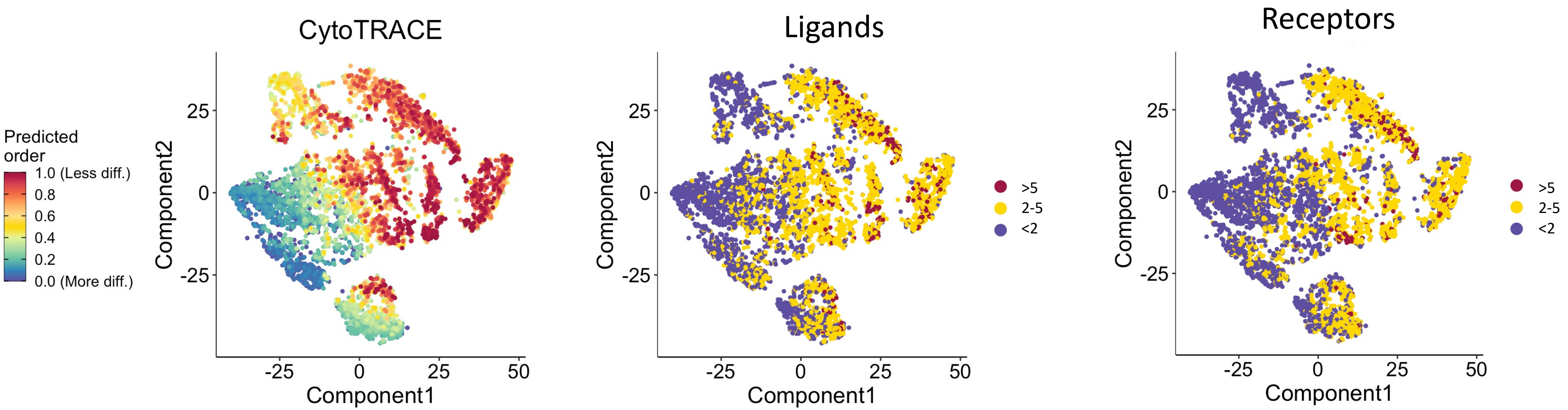

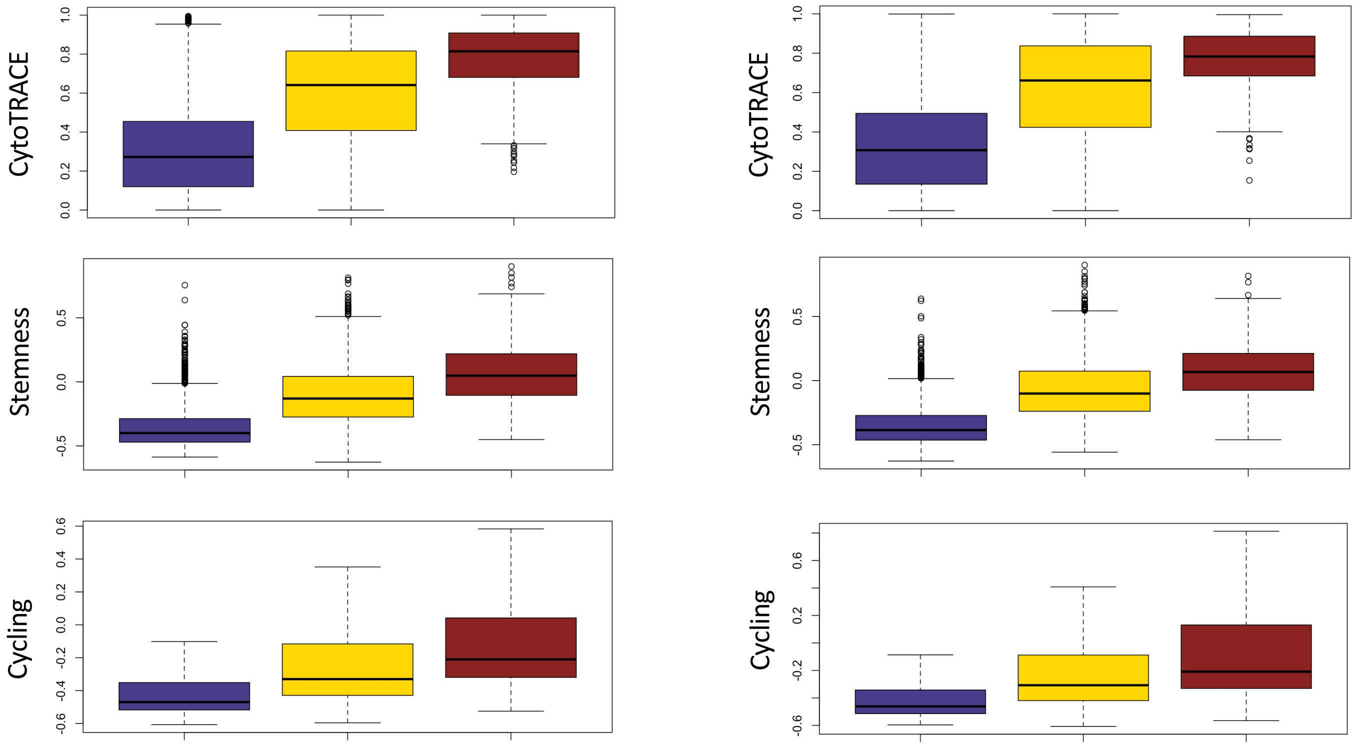

Supplemental Figure 3: Validating the developmental hierarchy of SHH medulloblastoma as a function of stromal activation. Top-left: t-SNE embedding of CYTOTRACE output, where less differentiated cells are coloured red and more differentiated cells are coloured blue. The same embedding is coloured by ligand (top-middle) and receptor (top-right) density of stroma-associates, suggesting “stroma-active” cells have greater developmental potential than “stroma-inactive” cells. Boxplots are used to compare stemness and cycling properties of cells expressing <2, 2-5, and >5 receptors and ligands, using both CYTOTRACE output and previously established “stemness” and “cycling” gene signatures.
